# Impact of the COVID-19 pandemic on international business travel and associated health issues: a survey of Japanese public companies

**DOI:** 10.1539/eohp.2025-0016

**Published:** 2025-11-21

**Authors:** Yayoi Tetsuou Tsukada, Ritsuko Okamura, Masahiro Yasutake

**Affiliations:** 1 Department of General Medicine and Health Science, Nippon Medical School, Tokyo, Japan; 2 Department of General Medicine, Nippon Medical School Musashi Kosugi Hospital, Kanagawa, Japan

**Keywords:** COVID-19, Japan, occupational health, overseas business travel, public companies

## Abstract

**Background:** The globalization of business has significantly increased the number of international business travelers (IBTs), yet their health issues remain inadequately studied. Short-term IBTs, who travel for less than 6 months, lack mandatory health checks under Japanese law, making it difficult to assess their health risks. The COVID-19 pandemic further complicated business travel, highlighting the need for enhanced health management strategies.

**Methods:**

A cross-sectional questionnaire survey was conducted among listed companies in Japan between September and December 2021. The survey targeted general affairs and human resources departments of 3,845 companies, yielding 251 valid responses (6.5% response rate). The questionnaire covered the necessity of business travel, health concerns before and after COVID-19, and expectations for occupational health support. Statistical analyses, including Pearson’s chi-square test and text mining, were performed to evaluate trends.

**Results:**

Before COVID-19, key health concerns included medical issues during travel (82.2%), infectious disease prevention (69%), and general health management (19.7%). Post-pandemic, priorities shifted to COVID-19 prevention, infectious disease control, and mental health support. Large companies emphasized psychological care, while smaller firms focused on infectious disease management. Business travel remained crucial for 85% of respondents, particularly for on-site guidance and sales.

**Conclusions:**

The pandemic underscored the need for comprehensive health management for IBTs, incorporating infection control, psychological support, and preventive care. As global travel resumes, companies must reassess health strategies to mitigate risks and ensure traveler well-being.

## Introduction

The globalization of economic and social activities has led to a marked increase in international business travelers (IBTs), with global figures rising from 642 million in 1980 to approximately 3.4 billion in 2019. In Japan, the number increased from 127,000 in 1964 to nearly 20 million in 2019^1,2)^. Business and professional travel accounts for 14% of all international tourist arrivals worldwide^3)^ and 12.3% in Japan^2)^.

IBTs face a myriad of challenges, including long-distance travel, jet lag^4,5,6)^, psychological burdens associated with job responsibilities and workloads^7)^, environmental and cultural differences, and limited access to medical care^8)^. However, the health issues experienced by expatriates are not as well understood^9,10)^. Additionally, the conflict between work and family life caused by transfers without family members has garnered attention^8)^. To date, reports on the health problems faced by IBTs have primarily focused on issues encountered during business trips, such as specific infectious diseases (eg, malaria, traveler’s diarrhea, sexually transmitted diseases) and travel-related trauma. Despite the high levels of physical and mental stress, there is a surprising paucity of reports on comprehensive medical care for IBTs. This gap underscores the need for more extensive and detailed research into the health impacts of frequent business travel.

The limited scope of comprehensive health studies on short-term IBTs may be due to the difficulties in tracking their health trends over brief periods. In Japan expatriate employees, who are assigned overseas for 6 months or longer, are tracked using visa applications; therefore, the actual number is known. Additionally, overseas resident officers need to perform medical checks before departing from their destination and after returning to Japan according to Japanese law. However, because short-term IBTs are not legally required to notify the Ministry of Foreign Affairs or undergo health checks according Japanese law (Industrial Safety and Health Act; Act No. 57 of 1972), it is impossible to determine their number or health status. Some reports use health insurance claim data of large global organizations to investigate health issues related to IBT. However, these studies have been confined to single organizations, which restricts the generalizability of the results^11,12)^.

The coronavirus disease 2019 (COVID-19) pandemic has severely impacted overseas business travel, with the number of IBTs dropping to 3.1 million in 2020 and 0.5 million in 2021^1)^. The COVID-19 pandemic disrupted overseas IBTs, including restrictions on overseas travel, polymerase chain reaction (PCR) testing, quarantine at entry and exit, differences in infection control measures from country to country, infection at the destination, and differences in the quality and systems of medical care.

As of September 2022, international business travel gradually resumed with the easing of pandemic-related restrictions. Safeguarding the health of IBTs has become increasingly critical, as unforeseen medical events abroad may jeopardize both employee well-being and corporate operations. Japan’s reliance on long-haul air travel — due to its geographic insularity — exacerbates the physical strain of international travel, with long flight durations and significant time zone changes. Concurrently, travel to nearby Asian countries — regions with persistent infectious disease risks — has increased. Despite these risks, pre-pandemic awareness of travel-related health among IBTs and employers remained low^13,14)^. Among Japanese IBTs, the rates of pre-travel medical consultations and vaccinations were notably low^15)^.

The persistence of pandemics may decrease travel frequency in certain sectors as online conferencing becomes more prevalent, reducing the necessity for physical travel. The challenges faced may also vary depending on the company size. Consequently, global companies are urged to reassess and enhance health management strategies for IBTs post-COVID-19 pandemic^16,17,18)^. The health issues faced by IBTs, who play a crucial role in the global economy, development, and growth, constitute a worldwide concern that cannot be addressed solely by individual companies. Understanding the trends in business travel and emerging health concerns is vital.

This study conducted a questionnaire survey to assess the trends and challenges associated with overseas business trips among publicly traded companies listed on the stock exchange in Japan.

## Methods

### Survey participants and definitions

This study was a cross-sectional online and off-line questionnaire survey conducted and was analyzed according to the American Association for Public Opinion Research Standard Definitions and based on mail surveys of unnamed persons^18)^. The target population was companies on the stock market that are listed on the Electronic Disclosure for Investors’ Network^19)^. This database is used as it is publicly available, covers all public companies, and is updated monthly. As departments with jurisdiction details were unavailable, the questionnaire was addressed to the health management division of the general affairs and human resources department. It was sent on September 1, 2021, and responses were accepted online (Google form) or by mail until December 31, 2021. Responses collected by mail were additionally entered into the online questionnaire after the deadline. We received 43 responses through the web and 214 responses via mail. The mailed responses were digitized into the web survey format by our research assistants. To ensure completeness, all online responses were made mandatory, resulting in no instances of missing data.

The processing of mailed responses for required fields was conducted as follows: for entries missing the industry category (n=2), we could deduce the industry for one entry based on the company name, while the other remained unclassified due to identification challenges. In the case of Question 11, which had one missing response (n=1), the absence was recorded as such. For Question 12, one response (n=1) was categorized under “other,” and for Question 13, three responses (n=3) were noted as “neither.” Involving the public in the design, implementation, reporting and dissemination program of the study was considered, but it was impossible to work with targeted companies during the pandemic.

In Japan, employers must provide a medical examination for workers dispatched overseas for 6 months or more. Consequently, within the context of health management, this study defines short-term IBTs as someone traveling for less than 6 months.

The questionnaire survey was designed to identify trends in corporate deployment during and after the COVID-19 pandemic, focusing on health management issues based on the result of the scoping review and previous reports concerning Japanese companies^14,15)^. This approach aimed to understand how health concerns have been handled and any shifts in focus due to the pandemic: (1) the necessity of overseas business travel in the company’s operations; (2) company size; (3) industry; (4) healthcare problems before the COVID-19 pandemic; (5) critical healthcare problems before the COVID-19 pandemic; (6) healthcare problems after the COVID-19 pandemic; (7) critical healthcare problems after the COVID-19 pandemic; (8) problems encountered in healthcare after the COVID-19 pandemic; (9) expectations of industrial physicians and travel clinics in the post-COVID-19 era; (10) number of trips taken before the COVID-19 pandemic; (11) destinations before the COVID-19 pandemic; (12) estimated number of trips after the COVID-19 pandemic; (13) estimated destinations after the COVID-19 pandemic; (14) importance of business travel after the COVID-19 pandemic; (15) importance of business travel after the COVID-19 pandemic; (16) importance of business travel after the COVID-19 pandemic and reasons; and (17) reasons or purposes for continuing to travel overseas for business after the COVID-19 pandemic (Supplement 1).

### Data analysis

The sample size for this study was calculated using the following formula^20)^:

$$n\;=\frac{\frac{z^{2}\;\times p\left(1-p\right)}{e^{2}}}{1+\left(\frac{z^{2}\times p\left(1-p\right)}{e^{2}N}\right)}$$

n=the required sample size (persons), N=the population size (persons), z=the confidence level (Z-score), p=the response rate (% decimal point), e=the tolerance error (%: decimal point).

In this survey, using a tolerance error of 5%, a confidence level of 95%, and a response rate of 50%, the optimal sample size was calculated to be 254 cases.

Categorical variables were expressed as crude frequencies and percentages. The importance of health issues before and after the pandemic, number of trips, destination, and business travel were examined using a Poisson frequency test for multiple responses and Pearson’s chi-square test for single answers to determine whether there was a difference in the proportions of these variables. The two-tailed Pearson’s chi-squared test was used to test for equality of proportions. Text mining analysis was conducted on the open-ended questionnaire responses, which focused on identifying specific healthcare challenges faced by short-term IBTs during the COVID-19 pandemic. Keywords were inductively extracted from respondents’ statements, and their frequencies were subsequently calculated.

For all analysis, a two-sided p≤0.05 was considered significant. All analyses were performed using JMP (version 14.02) statistical software (SAS Institute; Cary, NC, USA).

## Results

### Background of responding companies

**Fig. 1.**
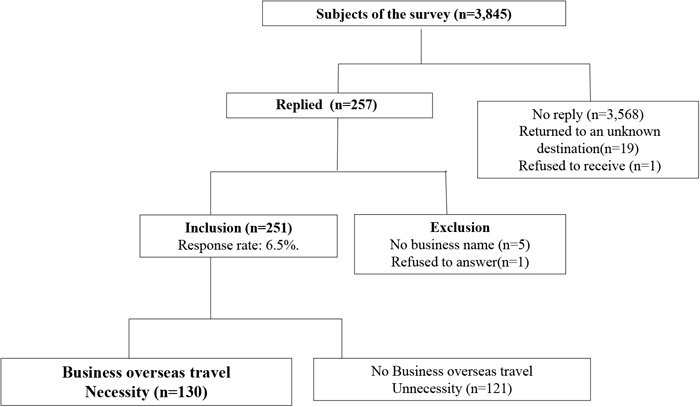
Questionnaire collection status

Among the 3,845 companies that received questionnaires, 19 returned them to an unknown address, and one refused to receive them. Of the 257 companies that responded, 5 did not indicate the company’s name, 1 declined to respond, and 251 provided valid responses (response rate: 6.5%; Figure 1). Of the companies that responded correctly, 130 (52%) indicated that business travel was necessary for their operations. Of these, 104 (80%) were large companies with 301 employees or more. Of the 121 companies that answered that business travel was unnecessary (121 companies), 68 (56%) were small- and medium-sized companies with 300 or fewer employees. Types of industries that replied unnecessarily were the manufacturing, wholesale/retail, and service (not elsewhere classified) industries (Table 1).

**Table 1.** The background of the responding companies

Necessity of Overseas Business Travel	Yes (%)	No (%)	Total (%)
**Total**	130 (51.6)	121 (48.4)	251 (100)
**Company size (number of employees)**			
>1,000	60 (24.0)	26 (10.0)	86 (34.4)
301–1000	44 (17.2)	27 (10.8)	71 (28.0)
101–300	18 (7.2)	41 (16.4)	59 (23.6)
1–100	8 (3.2)	27 (10.8)	35 (14.0)
**Type of Industry**			
Manufacturing industry	69 (27.5)	16 (6.4)	85 (33.9)
Wholesale and retail	15 (6.0)	27 (10.8)	42 (16.7)
Services (not elsewhere classified)	10 (4.0)	20 (8.0)	30 (12.0)
Telecommunications industry	10 (4.0)	18 (7.2)	28 (11.2)
Construction industry	9 (3.6)	9 (3.6%)	18 (7.2)
Real estate business, equipment leasing business	2 (0.8)	5 (2.0%)	7 (2.8)
Lodging, restaurant	1 (0.4)	6 (2.4%)	7 (2.8)
Transportation, postal service	2 (0.8)	5 (2.0%)	7 (2.8)
Unclassifiable industries	2 (0.8)	3 (1.2)	5 (2.0)
Lifestyle-related services, entertainment	2 (0.8)	3 (1.2)	5 (2.0)
Finance, insurance	2 (0.8)	3 (1.2)	5 (2.0)
Academic research, professional and technical services	1 (0.4)	3 (1.2)	4 (1.6)
Electricity, gas, heat supply, and water supply	2 (0.8)	1 (0.4)	3 (1.2)
Agriculture, forestry	1 (0.4)	1 (0.4)	2 (0.8)
Medical, social welfare	0 (0.0%)	1 (0.4)	1 (0.4)
Complex service business	1 (0.4)	0 (0.0%)	1 (0.4)
Mining, quarrying, gravel extraction	1 (0.4)	0 (0.0%)	1 (0.4)

Eighty-five percent of companies said that overseas travel will continue to be important or somewhat important in the post-COVID-19 era. The purpose of such trips was to provide technology and guidance on-site, as well as conduct business meetings and sales (Supplement 2).

### Number of yearly trips and destinations before and after the COVID-19 pandemic

Before the COVID-19 pandemic, employees from 42 companies (32.3%) had traveled more than 100 times per year, and employees from 64 companies (49.2%) had traveled between 10 and 100 times. However, after the COVID-19 pandemic, 57 companies (43.8%) responded that employee travel was difficult to predict, and only 11 companies (8.5%) had more than 100 cases of employees traveling for business up until September 2021. The results indicated that the larger the company, the greater the number of trips before the COVID-19 pandemic (Pearson’s p=0.001). However, there was no difference in the estimated number of trips according to company size after the COVID-19 pandemic. Regarding the share of destinations before and after the COVID-19 pandemic, China and Asia accounted for the largest share at 40%, followed by North America, Europe, and Russia. There were differences in travel destinations by company size and industry; however, no differences existed before and after the COVID-19 pandemic (Table 2).

**Table 2.** Annual number and destinations of business trips before and after COVID-19 pandemic

	Before	After
**(A) Number of Yearly Business Travels**		
>500	13 (10%)	1 (0.8%)
300–500	11 (8.5%)	1 (0.8%)
100–300	18 (13.8%)	9 (6.9%)
10–100	64 (49.2%)	22 (16.9%)
1–10	24 (18.5%)	34 (26.2%)
None		6 (4.6%)
Unpredictable		57 (43.8%)
**(B) Destinations**		
Africa	7 (2.8%)	7 (2.8%)
Europe/Russia	63 (25.1%)	58 (23.1%)
Pacific	13 (5.2%)	13 (5.2%)
China/Asia	127 (50.6%)	125 (19.8%)
Middle East	15 (6.0%)	14 (5.6%)
South America	13 (5.2%)	14 (5.6%)
North America	77 (30.7%)	77 (30.7%)

### Health issues for short-term business travel

**Fig. 2.**
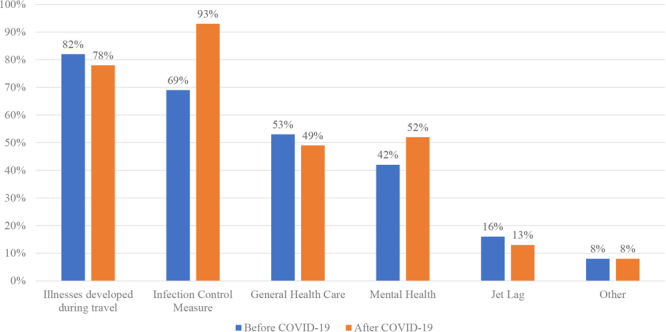
Health care issues of short-term business before and after COVID-19 pandemic (multiple answers)

Figure 2 shows the health issues associated with short-term business travel before and after the COVID-19 pandemic. The most common was “medical problems encountered during the business trip” (82.2%); followed by “coping with infectious diseases” (69%); and “general health management, such as lifestyle-related diseases” (25 companies, 19.7%). Numerous companies (54 companies, 42.5%) cited “medical problems encountered during business travel” as the most important concern (54 companies, 42.5%).

**Fig. 3.**
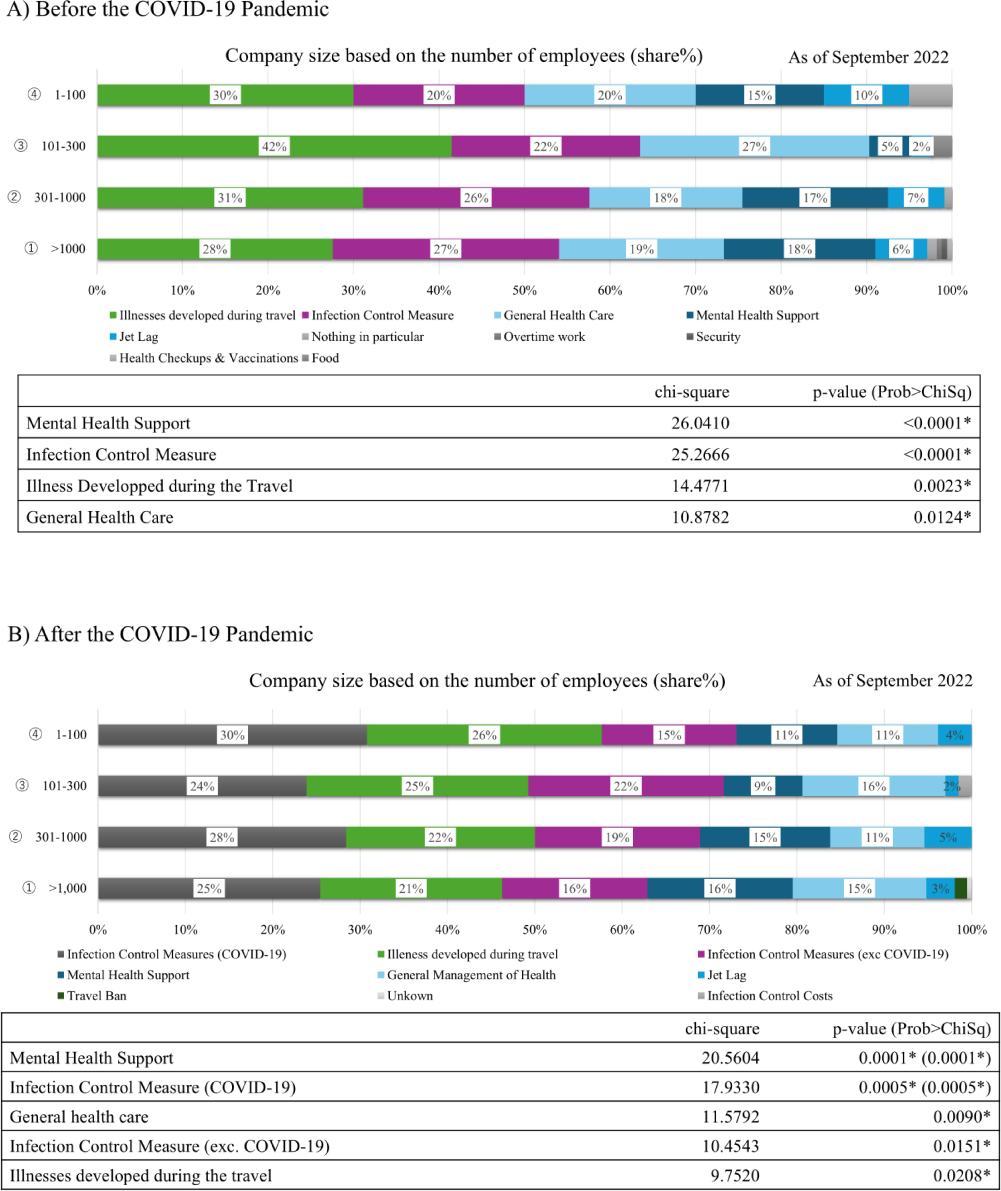
Health care issues of short-term overseas business travelers by company size

Based on company size, large companies (ie, companies with more than 300 employees) typically focus on “mental health support” and “prevention of infectious diseases.” However, small companies (ie, companies with 300 or fewer employees) commonly deal with “medical problems encountered during business travel” and “general illnesses,” such as lifestyle-related diseases. “General health management, such as lifestyle-related diseases” varies depending on the size of the company (Figure 3A).

However, after the COVID-19 pandemic, the essential items were “prevention of COVID-19 infection” (121 companies); “dealing with infectious diseases other than COVID-19” (83 companies); “medical problems encountered during business travel” (101 companies); “psychological support” (67 companies); and “general health management, such as lifestyle-related diseases” (63 companies), in that order (Figure 3B). Numerous companies cited “prevention of coronavirus infection” as the most critical factor. An analysis based on company size revealed that larger companies tended to prioritize “psychological support,” whereas smaller companies focused more on “COVID-19 infection control measures;” “general health management, such as lifestyle-related diseases;” “infection control measures against non-COVID-19 infectious diseases;” and “medical issues encountered during business travel” (p<0.001). We did not investigate differences across industries, as industries requiring overseas travel are predominantly in the manufacturing sector.

Numerous companies mentioned vaccines during a pandemic because the survey was conducted when vaccines were difficult to obtain. Others pointed to mental health concerns, especially during quarantine, a prompt examination system, and the sharing of updated and correct information. There were comments on post-return procedures and cluster measures, the lack of regular medicine, and healthcare because of the extended quarantine. Additionally, the industrial physician/travel clinic was expected to provide guidance, advice, and judgment regarding business travel, local medical care, mental support, and medical care using information and communications technology (ICT) as well as PCR tests (Table 3).

**Table 3.** Problem cases during a COVID-19 outbreak and expectations of industrial physicians and travel clinics

1. Specific examples of problems	
(A) Free comments	
**Responses (70 companies)**	
PCR testing and negative proof (convenience and cost)	11
Information sharing and provision	11
Guidance, advice, and judgment	9
Therapeutic drugs and vaccines (information and delivery system)	7
Medical care at the destination	5
Mental support	5
Online medical care	3
None	19
(B) Text mining analysis about specific health care challenges of business travellers during the COVID-19 pandemic	
**Responses (79 companies)**	
Travel ban	22
Preventative measures (prevention/vaccine related)	15
Mental health through isolation (isolation/mental)	9
Response to infected person response and local information	8
None in particular	7
General health issues (general health care/checkups)	5
Infection control in the workplace	3
(post-return employment procedures/workplace cluster measures)	
PCR test	2
2. Expectations of industrial physicians/travel clinics	
• Infection at the destination country. Obtain local medical information, secure hospital beds.	
• Isolation measures at the destination and return to Japan. Focus on psychological support during this period.	
• Return-to-work rules for those returning from abroad. Cluster measures in the workplace.	
• Lack of medicine taken regularly. General healthcare owing to prolonged quarantine.	
• Understanding local information before departure.	

## Discussion

This study conducted a questionnaire survey (Supplement 3) of listed companies to gain insight into the health issues of short-term IBTs and the impact of COVID-19 on short-term IBTs. The results indicated that prior to the emergence of the COVID-19 pandemic, the primary health concerns of short-term IBTs were general health issues, including illnesses that could develop during business travel, management of infectious diseases, and lifestyle-related diseases. Following the onset of the pandemic, the focus shifted toward the prevention of COVID-19 infections, as well as the management of other infectious diseases, the treatment of illness during travel, the provision of mental health support, and the maintenance of general healthcare. There were notable discrepancies in the healthcare priorities of large and small companies, as well as across different industries.

### Trends in overseas and Japanese travel

As COVID-19 restrictions were gradually lifted worldwide, international travel resumed. On September 4, 2022, the Japanese government lifted all landing restrictions previously imposed on travelers to Japan. Visa exemption arrangements resumed on October 11, 2022, and as of April 29, 2023, all travelers and returnees were no longer required to submit a negative COVID-19 test result or proof of vaccination^21)^. Consequently, international tourism rebounded significantly, with arrivals reaching 57% of pre-pandemic levels by July 2022. The number of international tourist arrivals nearly tripled (+172%) between January and July 2022 compared to the same period in 2021^1)^.

Although ICT-based alternatives, such as online conferencing, have become widely available, facilitating remote meetings and discussions, many business operations, including audits and on-site guidance, continue to necessitate in-person travel. While business travel has declined, it remains a crucial component of global business activities in the post-pandemic era.

### Health issues of short-term business travelers in the pre-COVID-19 pandemic period

Prior to the COVID-19 pandemic, only a few reports assessed the health problems of short-term international business travelers. In Japan, employers are legally obligated to conduct pre- and post-deployment medical examinations for those who have been sent overseas for 6 months or longer (Occupational Safety and Health Law). However, short-term overseas business travelers are not obligated to undergo health checkups related to their business trips, and the management of pre-trip health checkups is delegated to companies. Pre-travel health education, vaccination, and other measures for communicable diseases are optional. The number of short-term overseas travelers was greater than the number of long-term overseas travelers. However, it is difficult to identify and manage such health problems. Therefore, insufficient action has been taken to address this issue. According to a report in another country, business travelers are predominantly men, generally older, and seek pre-travel consultations largely on the advice of their employer^22)^.

Based on our survey, the most important health issue for overseas business travelers during the pre-pandemic period was the medical problems encountered during business travel. In Japan, work accident insurance and public insurance cover some cases, but only for limited cases. In cases where business travelers are not covered by appropriate insurance, companies may be obligated to assume a considerable portion of the resulting medical and emergency evacuation costs. The protection of business travelers’ health should be recognized as a core element of corporate social responsibility and an integral component of organizational risk management.

Infectious disease control, previously considered a secondary concern, emerged as the foremost challenge for companies during the COVID-19 pandemic. Prior to the pandemic, the importance of vaccination and pre-departure medical consultations had been acknowledged, and various initiatives targeting preventable infectious diseases among individual business travelers were reported. Compared to other types of travelers, business travelers tend to seek pre-travel healthcare closer to their departure dates and frequently decline recommended vaccinations, such as those for influenza, meningococcal disease, and hepatitis B^22)^. More recently, the rise of “last-minute travelers” — individuals who seek medical consultations immediately before departing on overseas assignments — has become a growing concern, creating logistical and clinical challenges for healthcare providers and occupational health systems^23)^. In the post-pandemic era, enhanced infection control measures are required, including up-to-date vaccinations and certification protocols. Survey results indicate that companies were compelled to adopt new practices during the pandemic, such as monitoring infection trends in destination countries, instituting return-to-work protocols for employees returning from abroad and implementing workplace cluster containment strategies. In response, Japan’s Ministry of Health, Labour and Welfare issued the Guidelines for New Influenza Preparedness in Enterprises and Workplaces, urging companies to establish advance response plans tailored to different phases of a pandemic^24)^.

In addition, before the COVID-19 pandemic, many respondents, primarily large companies, emphasized the importance of general healthcare, such as hypertension and diabetes. The relationship between business travel and lifestyle-related diseases has not yet been established. Previous studies reported that non-travelers were more likely to report poor/fair health compared with light travelers (1–6 nights per month), and with increasing travel, reaching 2.61 among extensive travelers (>20 nights per month). Compared with light travelers, the odds ratios for obesity were the highest among non-travelers and extensive travelers^25)^. Another study indicated stronger associations between the sum of domestic and international travel and body mass index, body fat percentage, and visceral adipose tissue in women than in men after accounting for age, exercise, and sleep. Based on the male sample population, international travel frequency has a greater influence on adiposity than summed (mostly domestic) travel^26)^.

Conversely, an analysis of 12,942 unique health risk appraisal records of United States employees of a multinational corporation indicated that international business travel was significantly associated with a lower body mass index and lower blood pressure^27)^. Generally, it has been noted that social jetlag, such as in shift workers, is associated with metabolic syndrome and cardiovascular events^28,29)^. To date, there has been no analysis of the number of business trips, obesity, and metabolism among the Japanese population. Further studies are required to clarify the relationship between short-term business trips and lifestyle-related diseases. Given the lack of opportunities for health checkups and increased cost burden for companies in developing diseases overseas, companies must adequately manage their general health.

Finally, psychological support should be provided. Several studies have reported the importance of emotional support for international business travelers^30,31)^. A survey on health management measures among business travelers who had travelled overseas for less than 6 months revealed that approximately 30% of respondents experienced mental health problems or work-related disasters^32)^. In addition, our study determined that psychological challenges were more common in larger firms. Further, our study found that the analysis based on company size revealed that larger companies tended to prioritize “psychological support” (p<0.001), whereas smaller companies focused more on “COVID-19 infection control measures” (p<0.001) and “general health management (p<0.001)” after the COVID-19 pandemic (Figure 3B). The correlation between company size and psychological stress has not been previously reported. There were no differences in the regions where the responding companies travelled but it is possible that the larger the company, the more important the duties and responsibilities of the IBTs. Additionally, unlike expatriates, IBTs who do not bring their families with them face the challenge of balancing work and family life, which can also be a source of stress. A significant correlation between the number of business trips and the difficulty of balancing work and family life has also been reported^33,34)^. The survey results indicate that during a pandemic, the psychological burden on business travelers is high owing to unexpected infection and isolation at the destination as well as differences in the medical system. ICT is helpful for workers abroad to share correct information and psychological support in a timely manner^35)^.

This study was conducted between September and December 2021, during a period of heightened uncertainty and anxiety due to the COVID-19 pandemic. It is plausible that the respondents’ perceptions of health risks and needs — particularly regarding infectious disease control and psychological support — were strongly influenced by the prevailing pandemic context. If a similar survey were conducted after the widespread stabilization of COVID-19, the prioritization of health concerns might differ. For instance, the relative emphasis on infection prevention may decline, whereas more enduring health issues such as chronic disease management or work–life balance may become more prominent. Thus, the findings should be interpreted in light of the temporally specific social and epidemiological landscape in which the data were collected.

Thus, global companies are required to consider general occupational health management and industrial hygiene related to overseas business travel^36,37)^. The International Society of Travel Medicine offers travel clinics, their members, and a network of physicians who can manage overseas business travelers. In addition, the main curriculum for training industrial physicians in Japan mandates health education for those sent overseas. As overseas business travel has resumed, it may be necessary to review the health management of business travelers.

### Limitations

The survey had a notably low responding rate of 6.5%. Possible reasons for this could include employees working remotely due to the pandemic and uncertainty regarding where the company mail should be delivered. However, considering that the appropriate sample size is 254 cases as mentioned before, we believe that, statistically, it accurately reflects the population.

Second, this survey was conducted from the perspective of managers; therefore, the actual health status of business travelers could not be ascertained. Subsequently, it is necessary to further investigate the actual health status of business travelers at companies with frequent overseas business trips. The current conclusions should be taken in light of these limitations. However, these findings provide a meaningful step in understanding the health concerns of short-term expatriates and the impact of COVID-19 on this demographic.

Third, the timing of the survey may have introduced response bias. Conducted in late 2021, during an active phase of the COVID-19 pandemic, the heightened awareness and concern regarding infectious diseases and mental health may have influenced the prioritization of health issues among respondents. Consequently, the findings may reflect transient pandemic-related concerns rather than stable, long-term health priorities of short-term business travelers.

## Conclusions

Considering the health issues of business travelers, it was revealed that accidents and illnesses during business trips, prevention of infectious diseases, general health management, and psychological support are essential, and that what is required differs depending on the size of the company. As overseas business travel has resumed, the health management of business travelers should be reviewed.

## Supplementary Material

Supplement 1

Supplement 2

Supplement 3

## Data Availability

The data presented in this study are available on request from the corresponding author. The data are not publicly available because all responses were provided in Japanese only.
