# Supplementary material for: Impact of the COVID-19 pandemic on international business travel and associated health issues: a survey of Japanese public companies
Source: Environ Occup Health Pract. 2025 Nov 21;7(1):2025-0016. doi: 10.1539/eohp.2025-0016 (PMC12738531; doi:10.1539/eohp.2025-0016)
Supplement: Supplementary file 1 — Supplement 1 [file eohp-7-2025-0016-s001.pdf]

Supplement 1. Survey of Health Care Issues for Short-Term Overseas Business Travelers in Post-Corona era

Q1: Does your business require overseas travel services?

☐ Necessary　　　　　　　Not necessary

If you selected "necessary" in Q1, please answer the following questions (Q2-Q13).

If you chose "not required," please answer questions A-C.

Q2. What were the health care needs of short-term international travelers in before the spread of coronavirus infection (multiple responses allowed)?

☐ Diseases that may occur during the travel period
☐ Prevention of infectious diseases
☐ Time difference
☐ Mental health support
☐ General health care, including lifestyle-related diseases
☐Other (　　　　　　　　　　　　　　　　　　　　　　　　　　　　　　　　　　　)

Q3. Which of these were particularly important to you? Please select one.

☐ Diseases that may occur during the travel period
☐ Prevention of infectious diseases
☐ Time difference
☐ Mental health support
☐ General health care, including lifestyle-related diseases
☐Other (　　　　　　　　　　　　　　　　　　　　　　　　　　　　　　　　　　　)

Q4: What are the future needs in terms of health care for short-term international travelers after the spread of coronavirus infection? (Multiple answers allowed)

☐ Coronavirus infection control
☐ Response to infectious diseases other than coronavirus
☐ Diseases that may occur during the travel period
☐ Time difference
☐ Mental health support
☐ General health care, including lifestyle-related diseases
☐ Other (　　　　　　　　　　　　　　　　　　　　　　　　　　　　　　　　　　)

Q5. which of these have become particularly important to you? Please choose one.

☐ Coronavirus infection control

☐ Response to infectious diseases other than coronavirus

☐ Diseases that may occur during the travel period

☐ Time difference

☐ Mental support

☐ General health care, including lifestyle-related diseases

☐ Other ( )

Q6. Please tell us about any particular problems that have arisen in the health care of short-term international travelers as a result of the spread of coronavirus infections.

Q7. In the future, what do you expect to see in the health care of short-term international travelers by industrial physicians and travel clinics?

Q8: Please provide an approximate number of your company's annual business travel abroad prior to the spread of coronavirus infection.

☐1-10 cases ☐ 10-100 cases ☐ 100-300 cases ☐ 300-500 cases ☐ 500 or more cases

Q9. What are your main travel destinations? (Multiple answers allowed)

☐ China and Asia ☐ North America
☐ Europe and Russia ☐ South America
☐ Middle East ☐ Pacific
☐ Africa Region

Q10. Please let us know the annual number of business travel abroad in the post-Corona period (forecast).

☐ Difficult to predict ☐ Will disappear
☐ 1-10 cases ☐ 10-100 cases ☐ 100-300 cases ☐ 300-500 cases ☐ 500 or more cases

Q11: What are the main destinations of the post-Corona period? (Multiple answers allowed)

☐ China and Asia ☐ North America
☐ Europe and Russia ☐ South America
☐ Middle East ☐ Pacific
☐ Africa Region

If you answered in Q10 that you will resume business overseas travel in the post-Corona period, what is your reason or purpose for continuing your business overseas travel? (Multiple answers allowed)

☐ On-site technical support and guidance ☐ Commercial talks and sales
☐ Internal meetings ☐ Market research
☐ Events and conferences ☐ Delivery, repair, inspection, repair
☐ Training
Other ( )

Q13: What is the importance of short-term overseas travel for overseas business in the post-Corona period?

☐ Will continue to be important ☐ Somewhat important ☐ Neither
☐ Not very important ☐ Not important
Reason

A: How many employees does your company have?

☐ 1-100 persons ☐ 101-300 persons ☐ 301-1000 persons ☐ 1,001 persons or more

B: What is your company's type of business?

☐ Mining, Quarrying, Gravel extraction
☐ Construction
☐ Manufacturing
☐ Electricity, gas, heat supply, and water supply
☐ Information and Communication
☐ Transportation, postal service
☐ Wholesale and retail
☐ Finance, Insurance
☐ Real estate, goods rental
☐ Academic research, professional and technical services
☐ Agriculture, Forestry
☐ Fishing
☐ Lodging, Restaurants
☐ Lifestyle-related services, recreation
☐ Education, Learning Support
☐ Medical, Welfare
☐ Complex service business
☐ Services (not elsewhere classified)
☐ Public affairs (excluding those classified elsewhere)
☐ Unclassifiable Industries

C: Please provide any other comments or concerns about health care for short-term business travelers.

Company Name:

Name of person in charge:

Thank you very much for your cooperation.
