# Supplementary material for: Impact of the COVID-19 pandemic on international business travel and associated health issues: a survey of Japanese public companies
Source: Environ Occup Health Pract. 2025 Nov 21;7(1):2025-0016. doi: 10.1539/eohp.2025-0016 (PMC12738531; doi:10.1539/eohp.2025-0016)

Supplement 2. Importance and purpose of overseas travel in the post-Corona era

A. Importance of International Travel

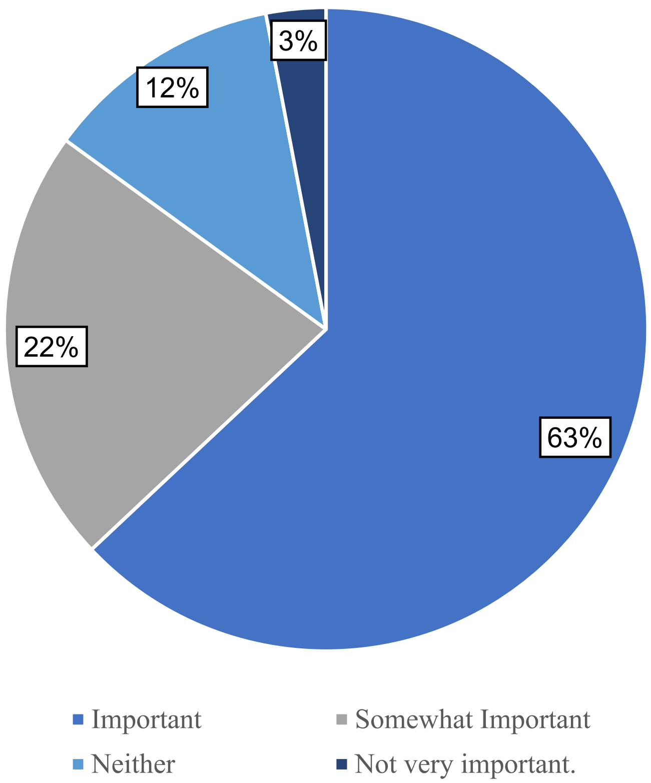

B. Purpose of International Travel

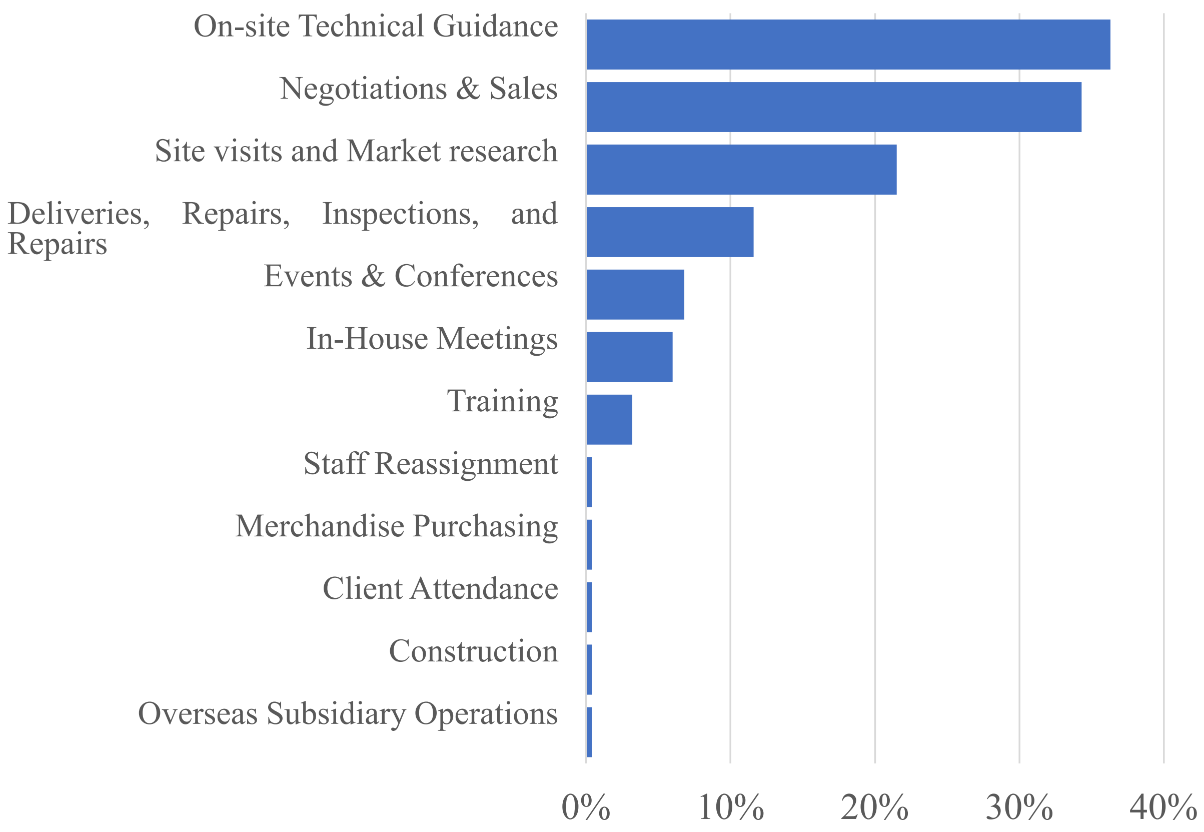

Supplement: Supplementary file 2 — Supplement 2 [file eohp-7-2025-0016-s002.pdf]
